# Liver and Spleen Stiffness Measurements for Assessment of Portal Hypertension Severity in Patients with Budd Chiari Syndrome

**DOI:** 10.1155/2019/1673197

**Published:** 2019-01-02

**Authors:** Elton Dajti, Federico Ravaioli, Antonio Colecchia, Giovanni Marasco, Amanda Vestito, Davide Festi

**Affiliations:** ^1^Department of Medical and Surgical Sciences (DIMEC), University of Bologna, Italy; ^2^Gastroenterology Unit, Borgo Trento University Hospital, Verona, Italy

## Abstract

**Aims:**

Budd-Chiari Syndrome (BCS) is a rare vascular disease of the liver caused by the obstruction of the hepatic venous outflow located from the small hepatic venules up to the entrance of the inferior vena cava (IVC) into the right atrium. Current prognostic indexes are suboptimal for an individual prognostic assessment and subsequent management of patients with BCS. Liver (LSM) and spleen (SSM) stiffness measurements are widely validated prognostic tools in hepatology, but the evidence in patients with BCS is limited. This paper describes LSM and SSM in patients with BCS and their correlation with clinical, biochemical, and ultrasound findings from the same patients.

**Methods:**

We investigated a case series of seven patients with BCS diagnosis and available LSM and SSM evaluated by transient elastography (TE). Biochemical, imaging, and endoscopic findings nearest to the TE evaluation were recorded. Clinical outcomes and BCS evolution were described for each patient. When available, repeated TE assessments were also recorded.

**Results:**

Patients with acute nonfulminant manifestation of BCS presented near-the-upper-limit values (75 kPa) of LSM and SSM, which often persist until the placement of a transjugular intrahepatic portosystemic shunt (TIPS). On the other hand, TE values were markedly lower in patients with compensated BCS. In some patients with repeated TE measurement years after TIPS placement, LSM had decreased to values of <10 kPa years. SSM changes in these patients were, however, less evident.

**Conclusions:**

Extremely elevated values of LSM and SSM are suggestive of BCS. The evaluation of both LSM and SSM by TE could help clinicians in the initial evaluation, risk stratification, and therapy response monitoring of patients with BCS.

## 1. Introduction

Budd-Chiari Syndrome (BCS) is defined as the obstruction of hepatic venous outflow located from the small hepatic venules up to the entrance of the inferior vena cava (IVC) into the right atrium [1]. To date, various prognostic indices (PIs) similar to the Child-Pugh classification, such as the Rotterdam [2] or Clichy Score [3], have been proposed to predict transplantation- (OLT-) free or overall survival in BCS patients [1]. However, these PIs have a relatively low predictive ability [4, 5] and are thus not suitable for an individual prognostic assessment and management [1]. Moreover, no specific noninvasive predictors of recanalization, portal hypertension (PH) severity, risk of decompensation event, or transjugular-intrahepatic-portosystemic shunt (TIPS) dysfunction are available. Yet such predictors would be very helpful for clinicians in monitoring BCS patients, in order to guide the therapeutic approach and evaluate its response.

Noninvasive tests (NITs), such as liver (LSM) and spleen (SSM) stiffness measurements, have been widely validated as accurate surrogates of PH [6] and its complications, such as high-risk esophageal varices [7, 8] or hepatic decompensation [9, 10], in patients with advanced chronic liver disease (ACLD). To our knowledge, only two studies [11, 12] have described LSM and its changes after endovascular treatment in BCS patients, suggesting that LSM reflects liver congestion rather than the fibrosis stage in these cases. On the other hand, SSM has not yet been described in this context. Studies exploring the prognostic role of such parameters in the context of vascular liver diseases, including BCS, are therefore needed [6].

This paper discusses a case series of patients diagnosed with BCS and their LSM and SSM by transient elastography (TE).

## 2. Case Presentations

Table 1 summarizes the main demographic, clinical, and radiological findings in our patient cohort with BCS.

1. A 34-year-old female with essential thrombocythemia (ET) presented to the emergency unit for body weight gain and increase in abdominal circumference; nocturnal pruritus and abdominal pain had been present since pregnancy. Doppler-ultrasound (US) established a diagnosis of BCS. An upper endoscopy (EGD) showed the presence of grade 1 esophageal varices (EV) and mild portal hypertensive gastropathy (PHG). TE evaluation at diagnosis showed LSM and SSM values of 75 kPa at both sides.
2. For the following two years, caudate lobe hypertrophy and a macronodular liver pattern (>2.5 cm) developed. Most importantly, ascites was resistant and intolerant to diuretic therapy, leading to the readmission of the patient for severe hyponatremia and massive pleural effusion. TIPS was thus placed.
3. In the following year, no liver-related complications were registered. EGD showed no signs of EV or PHG. At concomitant TE control, LSM was significantly reduced to 8 kPa, but high values of SSM persisted (65.2 kPa).
4. A 65-year old female with a history of persistent abdominal pain presented with a recent increase in abdominal circumference. The CT scan evaluation provided the diagnosis of BCS. No EV or PHG were shown at EGD. LSM and SSM were both 75 kPa. The hematological evaluation established an ET diagnosis.
5. In the following two years, EGD showed EV grade 1 and the patient developed recurrent episodes of ascites. At the last decompensating event, LSM and SSM were both 75 kPa. Accordingly, TIPS was placed and an evaluation for liver transplant was started. In the following month, the patient underwent OLT and no signs of BCS recurrence have been reported since then.
6. A 25-year-old male with an initial diagnosis of decompensated cryptogenic cirrhosis was referred for OLT evaluation due to severe PH with repeated bleeding episodes. In our unit, Doppler-US showed obliterated hepatic veins (HVs) and signs of severe PH. BCS diagnosis was confirmed at vena cavography. LSM and SSM were both 75 kPa.
7. At EGD controls, EV persisted (G1 with red signs) and PHG became severe. TIPS placement was attempted, but was unsuccessful. Consequently, in the following year, the patient developed numerous decompensating events. During one of these episodes, the bleeding could not be controlled, thus urgent OLT was attempted and successfully carried out.
8. A 52-year-old male was admitted to our unit with suspected BCS. Vena cavography and HV catheterization indicated ICV thrombosis. Liver biopsy highlighted macro-vesicular steatosis, thus ruling out the presence of significant fibrosis. EGD screening revealed the presence of grade 1 gastric varices.
9. In the following months, the patient presented several bleeding episodes which finally led to TIPS placement one year after BCS diagnosis. At TE evaluation before the intervention, LSM and SSM were both 75 kPa.
10. During a follow-up of over ten years, TIPS remained patent and no variceal bleeding or ascites occurred. At TE evaluation, 10-year post-TIPS placement, LSM was 7.3 kPa, while high values of 75 kPa for SSM were reported.
11. A 65-year-old female with BCS diagnosis presented the following medical history: long-standing Polycythemia Vera (PV); initial diagnosis of cryptogenic cirrhosis and idiopathic PH from diffuse nodular regenerative hyperplasia (NRH). Subsequently, serrated stenosis of right and left HVs were demonstrated. After percutaneous transluminal angioplasty (PTA) and TIPS placement, the patient remained compensated and was subsequently lost at follow-up.
12. During evaluation in our center: EGD showed mild PHG, but no EV; US described signs suggestive of advanced liver disease, minimal ascites and normal TIPS flow velocity. However, in the following year, TIPS was obstructed and subsequently compensated by recanalization of the paraumbilical vein. LSM and SSM at this time were both 75 kPa. No events of clinical decompensation were reported in the follow-up although EGD showed the development of grade 1 EV.
13. A 61-year-old female was referred to our center for mild and diffuse abdominal pain. Doppler-US described a narrowed and partly visible right HV. Caudate lobe hypertrophy and macronodules (>2 cm) were also described in the liver. No signs of portal hypertension were present. LSM and SSM were 34.8 kPa and 38.5 kPa, respectively.
14. At follow-up over six years later, no decompensating events had developed. A repeated TE evaluation showed slightly reduced LSM and SSM values.
15. A 23-year-old female with Behçet's disease was referred to our center for persistent fever. At CT-scan assessment, however, stenosed right and left HVs were described. Liver biopsy showed unspecific signs and ruled out significant fibrosis. At Doppler-US, no ascites was present, but a patent paraumbilical vein was observed, as well as a slight splenomegaly. EGD ruled out the presence of EV. LSM and SSM were 14.3 kPa and 47.2 kPa, respectively.
16. During follow-up, no ascites or other hepatic decompensation events developed. At TE control three years after diagnosis, LSM and SSM were 11.8 and 40 kPa respectively.

## 3. Discussion

The results of the present study suggest that LSM and SSM could help in stratifying for the risk of PH-related complications as well as monitoring therapy response in BCS patients.

LSM evaluation by TE represents the most studied and validated ultrasonography technique to noninvasively assess the degree of liver fibrosis [13]. This method allows assessing the biomechanical properties of the liver by recording the propagation of shear-waves caused by a mechanically induced impulse applied at the tissue surface [14]; the velocity is then converted to Young's modulus value expressed in kPa [15]. In healthy people, normal values of LSM range between 4.4 and 5.5 kPa [16, 17], whereas values ≥7.1 kPa indicate moderate fibrosis (F2) [13]. In ACLD patients, LSM also correlates with the hepatic venous pressure gradient (HVPG) [6, 8, 18], the gold standard of PH evaluation [19], and therefore it could be used to indirectly assess PH in such patients [6]. According to the last Baveno Consensus on PH, LSM values of >20-25 kPa can be used to noninvasively rule in the presence of clinically significant PH (CSPH) in ACLD patients [8]. However, LSM does not solely reflect liver fibrosis but can reflect many other conditions [13, 20–22], including liver congestion [22]. This is why LSM could play a role in the evaluation of BCS, since the hepatic venous flow obstruction found in these patients typically leads to liver congestion and a post-sinusoidal form of PH [6, 23]. This hypothesis is in fact supported by initial data from murine models [24] and two studies that performed LSM in BCS patients [11, 12].

SSM is another more recent noninvasive method that can assess the degree of portal hypertension. In fact, SSM reflects the congestion and other structural changes [25, 26] occurring in the spleen as direct consequences of the increased portal pressure, irrespectively of its cause [6]. Therefore, it is considered a direct and more suitable surrogate of PH than LSM, with consistent evidence showing a better performance of SSM in the prediction of clinically significant PH (CSPH) [27, 28] or EV [29] in patients with advanced chronic liver disease (ACLD). However, to date no data are available on SSM values in BCS patients.

Based on our series of BCS patients, we believe that the implementation of LSM and SSM in the assessment of BCS patients has several benefits. Firstly, patients with acute nonfulminant manifestation of BCS present with severe liver congestion and consequent markedly increased portal pressure, as reflected by near-the-upper-limit values of LSM and SSM (75 kPa). Such values are very uncommon in other liver diseases, such as ACLD or other conditions that lead to hepatic congestion [30, 31]. For instance, out of 643 ACLD patients included in one of our previous studies [7], only three patients presented both LSM and SSM at 75 kPa. Therefore, extremely high values of both LSM and SSM could be very suggestive of BCS in a decompensated patient with unclear liver etiology or with an inconclusive first ultrasound exam. This information could lead clinicians to request further imaging studies to confirm BCS diagnosis.

Moreover, TE values found at diagnosis can stratify the severity of BCS disease. For instance, all patients (1-3) with high LSM and SSM values at diagnosis developed recurrent decompensating events and underwent TIPS placement or OLT within a few years. Maximum TE values were maintained or found also before TIPS placement, thus confirming the findings of Mukund et al., who reported median values of 75 kPa before such intervention [11]. In contrast, the markedly lower TE values at diagnosis found in patients 6 and 7 were associated with milder chronic forms of BCS presentation; in fact, these patients did not develop events of clinical decompensation during follow-up and did not require invasive interventions, such as TIPS.

Our series thus presents new and very promising results regarding TE as a prognostic tool in BCS patients. This is the first study report that shows LSM and SSM values at BCS diagnosis in patients with different disease severity. The only two previous studies [11, 12] evaluating LSM in these patients were limited to its evaluation in patients with severe BCS and only before (and after) TIPS placement. Furthermore, values at TE evaluation seem to correspond to the degree of liver congestion and PH, as they clearly differ between patients with and without decompensated disease (patients 1-4 vs 6-7). Of note, the SSM values in patients 6 and 7 were below our previously described cut-off (54 kPa) for the prediction of hepatic decompensation in ACLD patients [10]. We believe that the measurement at baseline of both these parameters could better stratify the risk of PH-related complications and help clinicians to identify patients with more severe PH, who are more prone to recurrent decompensating events and require earlier TIPS placement or OLT. TE evaluation could therefore at least improve the prognostic information provided by the currently available PIs.

Whether LSM or SSM is the more informative parameter, it is still to be determined. We found a discrepancy between LSM and SSM values only in the two compensated patients (patients 6 and 7), due to higher LSM values in patient 6 (34.8 vs 14.3 kPa) and higher SSM values in patient 7 (38 kPa vs 47.2 kPa). However, clinical data, such as the presence of splenomegaly and periumbilical vein recanalization, suggested a higher portal pressure in patient 7, which could justify higher SSM (47.2 kPa). In patient 6, on the other hand, LSM could be related to a modest and heterogenous liver congestion, which is common in BCS patients and could justify our results. In fact, in this patient only the right HV was occluded, whose drainage area corresponded to the side where the LSM was performed. This suggests that SSM could be a more accurate predictor of disease severity, as it reflects PH directly and may not be influenced by the heterogeneity of BCS.

Response to therapy is another fundamental point to consider after BCS diagnosis. Doppler-US is the preferred noninvasive tool to monitor the dynamic changes in the level of obstruction over time/after therapy [1]; however, it lacks sensitivity in evaluating short- and long-term outcomes after therapy [32]. To date, two papers [11, 12] have evaluated short-term changes in LSM after derivative therapy in BCS patients. Mukund et al. [11] showed for the first time that LSM, assessed by TE, significantly decreased 24 hours after the endovascular intervention, but no correlation between changes in LSM and the pressure gradient was found. Later on, Wang et al. [12] confirmed that LSM, evaluated by share-wave elastography, significantly decreased two days after balloon angioplasty. The authors also demonstrated that before-treatment LSM values significantly correlated with hepatic venous gradient, but not with liver fibrosis degree, proposing LSM as a sensitive NIT to monitor hemodynamic changes after therapy in BCS patients. However, the follow-up period in these two studies was short (up to six months) and the relationship between LSM changes and clinical outcomes after treatment was not investigated.

In the present series, in most of the patients LSM became lower after therapy. Considering the retrospective design of the study, TE was not systematically repeated at scheduled time-points (i.e. 1 day, 3 months) after TIPS, thus our results are not comparable to the above-mentioned studies. However, we described for the first time LSM and SSM values for up to ten years after TIPS implementation. Most importantly, LSM not only decreased after TIPS placement in patients 1 and 4, but its values were also below the threshold of 10 kPa for ACLD. In our view, this is an important result, as it could show how liver parenchyma, and therefore liver function, is preserved in some BCS patients. These are the patients that mostly benefit from derivative interventions such as TIPS, and being able to identify them through repeated TE evaluations could be very helpful for clinicians.

On the other hand, a lack of decreasing LSM might help identify subjects who remain at risk of decompensation. This could reflect obstruction or the suboptimal efficacy of TIPS (as in patient 7), but most importantly, it could also reveal the “real” BCS-related cirrhotic patients (i.e., persistently high LSM values despite patent TIPS). Liver cirrhosis is often improperly used or erroneously diagnosed in BCS patients (i.e., patients 3 and 5), mostly due to the presence of US signs that are not unique to with this condition, such as caudate lobe hypertrophy and nodularity. Identifying BCS-related cirrhotic patients would be beneficial in selecting the best candidates for OLT, as a risk of decompensation in these patients is correlated with liver function impairment, rather than congestion.

As far as SSM is concerned, a slight decrease due to spleen decongestion may be observed after TIPS placement (i.e., patient 1) or anticoagulation (i.e., patient 6 and 7). However, these changes are less evident than the rapid decrease described for LSM as a result of liver decongestion. Persistent SSM values could reflect the long-standing structural changes that occur in the spleen due to PH development [25], together with the fact that there is still a risk of PH-driven complications in these patients [1]. However, the role of SSM changes after TIPS placement still needs to be clarified, also in the context of ACLD.

In conclusion, we believe that TE will soon become a valid prognostic tool in the context of vascular liver diseases, just as it is today for ACLD patients of viral etiology. At BCS diagnosis, LSM and especially SSM could stratify for PH severity and better identify patients that require TIPS placement. However, little information on the underlying chronic liver disease can be obtained, as LSM in these patients reflects the congestion rather than the fibrosis of the liver. Moreover, persistently high TE values despite derivative therapy could reveal the patients with real ACLD and liver function impairment; in these patients, OLT should be considered. Future multicentre studies in larger cohorts are highly encouraged in order to fully explore the potential of NITs in BCS.

## Figures and Tables

**Table 1:** Main demographic, clinical, and radiological findings in our patient cohort with BCS.

**Patient **	**Sex, Age**	**Etiological/Risk Factors**	**Prognostic Index (PI) at diagnosis**				**Outflow Obstruction;**	**Treatment**	**Time point of TE**	**LSM (kPa)**	**SSM (kPa)**	**Events after TE evaluation**
			**MELD-Na**	**Child-Pugh**	**Rotterdam Score** ^**#**^	**Clichy ** **S** **c** **o** **r** **e** ^§^						
1.	F, 34	ET, JAK2-	10	7	1.11	4.7	2/3 HVs fibrotic, thrombosis of portal, mesenteric and splenic vein	VKA	At diagnosis	75	75	Refractory ascites, TIPS
								TIPS	1 year after TIPS	8	65	No CD

2.	F, 65	ET, JAK2+	13	6	1.15	5.2	Complete thrombosis of all HVs	VKA	At diagnosis	75	75	Recurrent CD events TIPS, OLT
								TIPS	Before TIPS placement	75	75	
								OLT				

3.	M, 25	None	13	5	0.11	3.3	2/3 HVs interrupted, 1 partially thrombosed	NSBB	At diagnosis	75	75	Several CD events, OLT

4.	M, 52	PV, JAK2-	12	5	0.10	4.9	IVC thrombosis	NSBB VKA	Before TIPS placement	75	75	Single episode of HE after TIPS
								TIPS	10 years after TIPS	7.3	75	

5.	F, 65	PV, JAK2+	10	6	1.17	4.8	TIPS occluded	VKA	TIPS occlusion	75	75	Patency of paraumbilical vein, EV development
								TIPS				

6.	F, 61	None	7	5	0.02	3.2	1/3 HVs obstructed	VKA	At diagnosis	34.8	38.5	No CD
									1 year after diagnosis	26.6	35.2	

7.	F, 23	Behçet's disease	8	5	0.03	3.1	2/3 HVs partially obstructed	VKA	At diagnosis	14.3	47.2	No CD
									3 years after diagnosis	11.8	40	

#Rotterdam Score: 1.27 x encephalopathy + 1.04 x ascites + 0.72 x prothrombin time + 0.004 bilirubin (*µ*mol/L).

§Clichy Score: ascites score × 0.75 + Pugh score × 0.28 + age × 0.037 + creatinine × 0.0036 (*µ*mol/L).

CD: clinical decompensation; ET: Essential Thrombocythemia; EV: esophageal varices; F: female; HV: hepatic vein; LSM: liver stiffness measurement; M: male; NSBB: nonselective beta-blocker; OLT: orthotopic liver transplantation; PV: Polycythemia Vera; SSM: Spleen Stiffness Measurement; TE: Transient Elastography; TIPS: transjugular intrahepatic portosystemic shunt; VKA: vitamin K antagonists.

## Data Availability

The data used to support the findings of this study are included within the article.

## References

[1] European Association for the Study of the Liver (2016). EASL clinical practice guidelines: vascular diseases of the liver. *Journal of Hepatology*.

[2] Murad S. D., Valla D.-C., de Groen P. C. (2004). Determinants of survival and the effect of portosystemic shunting in patients with Budd-Chiari syndrome. *Hepatology*.

[3] Langlet P., Escolano S., Valla D. (2003). Clinicopathological forms and prognostic index in Budd-Chiari syndrome. *Journal of Hepatology*.

[4] Rautou P., Moucari R., Escolano S. (2009). Prognostic Indices for Budd–Chiari Syndrome: Valid for Clinical Studies but Insufficient for Individual Management. *American Journal of Gastroenterology*.

[5] Sakr M., Abdelhakam S. M., Elsayed S. A. (2017). Validation of prognostic indices in egyptian budd-chiari syndrome patients: A single-center study. *World Journal of Gastroenterology*.

[6] Berzigotti A. (2017). Non-invasive evaluation of portal hypertension using ultrasound elastography. *Journal of Hepatology*.

[7] Colecchia A., Ravaioli F., Marasco G. (2018). A combined model based on spleen stiffness measurement and Baveno VI criteria to rule out high-risk varices in advanced chronic liver disease. *Journal of Hepatology*.

[8] European Association for Study of Liver (2015). EASL-ALEH Clinical Practice Guidelines: non-invasive tests for evaluation of liver disease severity and prognosis. *Journal of Hepatology*.

[9] Kim B. K., Han K., Park J. Y. (2010). A Liver Stiffness Measurement-Based, Noninvasive Prediction Model for High-Risk Esophageal Varices in B-Viral Liver Cirrhosis. *American Journal of Gastroenterology*.

[10] Colecchia A., Colli A., Casazza G. (2014). Spleen stiffness measurement can predict clinical complications in compensated HCV-related cirrhosis: A prospective study. *Journal of Hepatology*.

[11] Mukund A., Pargewar S. S., Desai S. N., Rajesh S., Sarin S. K. (2017). Changes in Liver Congestion in Patients with Budd–Chiari Syndrome following Endovascular Interventions: Assessment with Transient Elastography. *Journal of Vascular and Interventional Radiology*.

[12] Wang H., Shi H., Cheng J. (2018). Real-time shear wave elastography (SWE) assessment of short- and long-term treatment outcome in Budd-Chiari syndrome: A pilot study. *PLoS ONE*.

[13] Dietrich C., Bamber J., Berzigotti A. (2017). EFSUMB Guidelines and Recommendations on the Clinical Use of Liver Ultrasound Elastography, Update 2017 (Long Version). *Ultraschall in der Medizin - European Journal of Ultrasound*.

[14] Bamber J., Cosgrove D., Dietrich C. F. (2013). EFSUMB guidelines and recommendations on the clinical use of ultrasound elastographypart 1: basic principles and technology. *Ultraschall in der Medizin / European Journal of Ultrasound (UiM/EJU)*.

[15] Sandrin L., Tanter M., Gennisson J.-L., Catheline S., Fink M. (2002). Shear elasticity probe for soft tissues with 1-D transient elastography. *IEEE Transactions on Ultrasonics, Ferroelectrics and Frequency Control*.

[16] Colombo S., Belloli L., Zaccanelli M. (2011). Normal liver stiffness and its determinants in healthy blood donors. *Digestive and Liver Disease*.

[17] Roulot D., Costes J.-L., Buyck J.-F. (2011). Transient elastography as a screening tool for liver fibrosis and cirrhosis in a community-based population aged over 45 years. *Gut*.

[18] Song J., Ma Z., Huang J. (2018). Comparison of three cut-offs to diagnose clinically significant portal hypertension by liver stiffness in chronic viral liver diseases: a meta-analysis. *European Radiology*.

[19] Bosch J., Abraldes J. G., Berzigotti A., García-Pagan J. C. (2009). The clinical use of HVPG measurements in chronic liver disease. *Nature Reviews Gastroenterology & Hepatology*.

[20] Arena U., Vizzutti F., Corti G. (2008). Acute viral hepatitis increases liver stiffness values measured by transient elastography. *Hepatology*.

[21] Millonig G., Reimann F. M., Friedrich S. (2008). Extrahepatic cholestasis increases liver stiffness (FibroScan) irrespective of fibrosis. *Hepatology*.

[22] Colli A., Pozzoni P., Berzuini A. (2010). Decompensated chronic heart failure: Increased liver stiffness measured by means of transient elastography. *Radiology*.

[23] De Gottardi A., Berzigotti A., Buscarini E., García Criado A. (2018). Garc*φ*a Criado, Ultrasonography in Liver Vascular Disease. Ultraschall der Medizin. *European Journal of Ultrasound*.

[24] Simonetto D. A., Yang H.-Y., Yin M. (2015). Chronic passive venous congestion drives hepatic fibrogenesis via sinusoidal thrombosis and mechanical forces. *Hepatology*.

[25] Mejias M., Garcia-Pras E., Gallego J., Mendez R., Bosch J., Fernandez M. (2010). Relevance of the mTOR signaling pathway in the pathophysiology of splenomegaly in rats with chronic portal hypertension. *Journal of Hepatology*.

[26] Kondo R., Kage M., Iijima H. (2018). Pathological findings that contribute to tissue stiffness in the spleen of liver cirrhosis patients. *Hepatology Research*.

[27] Colecchia A. (2012). Measurement of spleen stiffness to evaluate portal hypertension and the presence of esophageal varices in patients with HCV-related cirrhosis. *Gastroenterology*.

[28] Song J., Huang J., Huang H., Liu S., Luo Y. (2018). Performance of spleen stiffness measurement in prediction of clinical significant portal hypertension: A meta-analysis. *Clinics and Research in Hepatology and Gastroenterology*.

[29] Manatsathit W., Samant H., Kapur S. (2018). Accuracy of liver stiffness, spleen stiffness, and LS-spleen diameter to platelet ratio score in detection of esophageal varices: Systemic review and meta-analysis. *Journal of Gastroenterology and Hepatology*.

[30] Colecchia A., Marasco G., Ravaioli F. (2016). Usefulness of liver stiffness measurement in predicting hepatic veno-occlusive disease development in patients who undergo HSCT. *Bone Marrow Transplantation*.

[31] Taniguchi T., Ohtani T., Kioka H. (2018). Liver Stiffness Reflecting Right-Sided Filling Pressure Can Predict Adverse Outcomes in Patients With Heart Failure. *JACC: Cardiovascular Imaging*.

[32] Boozari B. (2008). Ultrasonography in patients with Budd-Chiari syndrome: diagnostic signs and prognostic implications. *Journal of Hepatology*.

